# Differentiating central fever from infectious fever in intracerebral haemorrhage

**DOI:** 10.1136/svn-2025-004441

**Published:** 2025-08-27

**Authors:** Felix Hess, Enayatullah Baki, Julian McGinnis, Tun Wiltgen, Hannah Scholz, Kathleen Bernkopf, Gerhard Schneider, Jan S Kirschke, Dominik Sepp, Bernhard Hemmer, Silke Wunderlich, Mark Mühlau

**Affiliations:** 1 Department of Neurology, School of Medicine and Health, Technical University of Munich, Munchen, Germany; 2 Institute for AI in Medicine, Technical University of Munich, Munich, Bavaria, Germany; 3 TUM-Neuroimaging Center, School of Medicine and Health, Technical University of Munich, Munich, Germany; 4 Department of Anesthesiology and Intensive Care Medicine, School of Medicine and Health, Technical University of Munich, Munich, Germany; 5 Department of Diagnostic and Interventional Neuroradiology, School of Medicine and Health, Technical University of Munich, Munich, Germany

**Keywords:** Stroke, Hemorrhage

## Abstract

**Background:**

In addition to infectious fever, stroke-related disturbances in thermoregulation, referred to as central fever, are frequently observed in patients with stroke, particularly in those with intracerebral haemorrhage (ICH). Rapid identification of the underlying cause of fever is crucial for treatment decisions. This study aims to identify clinical, laboratory and radiological parameters that differentiate central fever from infectious fever in patients with ICH.

**Methods:**

We included 547 ICH patients in this retrospective, single-centre cohort study. Fever was defined as a body temperature exceeding 38.3°C for at least 2 consecutive days. Central fever was characterised by the absence of an infection diagnosis, cultured pathogens and any other identified cause of fever. CT scans were assessed visually and with a 3D nn-UNet for segmentation and subsequent quantification of all ICH components. Voxel-based lesion-symptom mapping was performed to identify lesion locations related to central fever. Univariate analyses and multiple logistic regression were conducted.

**Results:**

Fever occurred in 213 patients: 54 with central fever, 156 with infectious fever and 3 with other causes. Central fever was linked to higher scores on the National Institutes of Health Stroke Scale and worse outcomes (p<0.01). It had an earlier onset (median day 2 (1–3) vs 6 (4–9) p<0.01) and was more frequent in patients with lesions affecting the left midbrain and hypothalamic region (p<0.01). In contrast, infectious fever was associated with higher levels of infectious parameters (ie, C reactive protein, procalcitonin and leucocyte count). Its early onset (p<0.001) and affection of the left hypothalamic region (OR=9.7 (1.6 to 58.837), p=0.013) emerged as independent predictors of central fever.

**Conclusions:**

Early onset and hypothalamic involvement are the strongest indicators of central fever, which may help guide evidence-based treatment decisions for patients with fever following ICH.

**WHAT IS ALREADY KNOWN ON THIS TOPIC**

**WHAT THIS STUDY ADDS**

- We identified the day of fever onset and involvement of the hypothalamic region as key differentiators between central and infectious fever in patients with intracerebral haemorrhage.

**HOW THIS STUDY MIGHT AFFECT RESEARCH, PRACTICE OR POLICY**

- Our findings may contribute to a more reliable identification of central fever and thereby support evidence-based treatment decisions for patients with intracerebral haemorrhage.

## Introduction

 Fever is a common symptom in patients with acute stroke. Infections such as urinary tract infections and pneumonia—conditions to which stroke patients are especially vulnerable—are the most frequent causes of fever.^1–4^ Alternatively, fever may result from stroke-related disturbances of central thermoregulation, known as central fever.^5–8^ Rapid and reliable identification of the fever’s cause is crucial in clinical practice, as hyperthermia can significantly hinder the recovery from acute brain injury.^9–11^ Early detection and treatment of nosocomial infections with antibiotics are essential to prevent life-threatening septic complications. On the other hand, rapid and accurate identification of central fever could lead to a more targeted use of antibiotics, helping mitigate the critical development of antibiotic resistance.^12^

Patients with intracerebral haemorrhage (ICH) appear to be a subgroup of stroke patients in whom central fever occurs disproportionately often.^6 13^ However, our knowledge of ICH-related central fever is limited, as previous studies on central fever often involved heterogeneous patient populations with different stroke subtypes or multiple neurological conditions (eg, subarachnoid haemorrhage and primary brain tumours).^6 7 13^ The few studies specifically addressing the occurrence of central fever in patients with ICH found high frequencies of central fever and an association with poor outcomes but did not identify consistent differentiators between central and infectious fever.^14 15^ Our retrospective single-centre study aims to identify clinical, laboratory and radiological indicators of central fever in patients with ICH to distinguish it from infectious fever.

## Methods

### Patient selection

This study was performed in line with the principles of the Declaration of Helsinki. All patients admitted to our university hospital with the diagnosis of spontaneous ICH (I61.0–61.9; International Classification of Diseases, Tenth Revision) between January 2017 and December 2022 were screened for the inclusion criteria: (1) admission within 72 hours after symptom onset, (2) at least three documented measurements of body temperature for at least 3 consecutive days and (3) CT performed within 24 hours after admission. Patients with incomplete datasets due to missing CT scans or insufficient documentation of body temperature were excluded. The online supplemental material includes a flowchart of the selection process (online supplemental figure 1).

### Temperature assessment and classification of fever

Core temperature assessment using a bladder probe serves as the gold standard for temperature measurement in our ICU and stroke unit. For patients in whom a urinary catheter was not indicated (17% of the study cohort), temperature was measured via the ear canal instead.

A trained investigator (HS) collected data on body temperature measurements and potential causes of fever by reviewing medical reports and daily clinical documentation (progress notes) from ICU and stroke unit patients. Specifically, the investigator extracted information on infection diagnoses established by treating physicians as well as results from infection diagnostics, including imaging reports (eg, chest X-rays), urine tests, microbiological analyses and laboratory results. Additionally, reports and progress notes were reviewed to identify clinical signs of infection as well as other possible causes of fever, such as drug reactions, rheumatic diseases, tumours, thrombosis or pneumonitis, as considered by the treating physicians.

Fever was defined by a body temperature exceeding 38.3°C for at least 2 consecutive days.^6 7^ Central fever was characterised by the absence of an infection diagnosis, the absence of cultured pathogens and any other identified cause of fever.^6 7^ Classification of fever type was performed by two blinded investigators (FH and EB), who categorised fever as infectious, central, of other causes or uncertain. Therefore, infection diagnoses were evaluated based on the criteria for nosocomial infections of the Centers for Disease Control and Prevention (CDC), National Healthcare Safety Network (NHSN), V. 01/2025.^16^ Fever was classified as infectious if there was a diagnosis by the clinical team, fulfilment of CDC/NHSN criteria for nosocomial infection, or growth of a pathogenic organism in cultures. Fever was classified as caused by another defined cause if supported by documented evidence in medical reports. In cases where none of these criteria was met, fever was deemed central.

Both investigators had an overlap in their ratings of more than 90%. In cases of differing assignments or when the fever cause was deemed uncertain by at least one investigator (9.6%), both investigators reached a consensus by discussion; if uncertainty remained, the fever was classified as infectious (N=5, 2.5%). Patients with causes of fever other than central or infectious were excluded from further analysis.

### Clinical and laboratory variables

We collected information on demographic variables and cardiovascular risk factors. Stroke severity at the time of admission was assessed with the National Institutes of Health Stroke Scale (NIHSS) and functional outcome at discharge with the modified Rankin Scale. In cases of fever, we evaluated the peak levels of the infection parameters leucocyte count, C reactive protein (CRP) and procalcitonin (PCT) during the whole fever period and on the first day of fever. Additionally, we gathered information regarding the day of fever onset in relation to ICH symptom onset, peak temperature, the duration of fever episodes, the response to antipyretic treatment and septic courses based on Systemic Inflammatory Response Syndrome (SIRS) as well as Sequential Organ Failure Assessment (SOFA) criteria.

### Imaging

Besides deep-learning-based lesion segmentation and Voxel-Based Lesion-Symptom Mapping (VLSM) (see below), CT scans were screened visually for imaging features associated with ICH potentially indicating broader impacts beyond the lesion’s location, such as affected hemisphere (right, left or infratentorial) and brain region (deep vs lobar). Additionally, two blinded investigators (FH and DS) evaluated signs of space-occupying effects, that is, brainstem compression (graded as 0: none, 1: only deformity or shift, 2: CSF at midbrain or pons level completely obliterated and 3: visible oedema in the midbrain, pons or medulla near the site of compression) and midline shift (maximum distance from midline in mm).

#### Lesion segmentation

For precise and reliable segmentation of all components of ICH, we trained three different 3D nn-UNet models.^17 18^ Single-class 3D nn-UNet models were trained and employed for the segmentation of parenchymal haemorrhage (PH, training set N=160, test set N=40, dice score 0.92±0.19) and perihaematomal oedema (training set N=80, test set N=20, Dice score 0.86±0.08). For the quantification of intraventricular haemorrhage (IVH) in each compartment of the ventricular system (lateral, third and fourth ventricles), a multiclass 3D nn-UNet was trained (training set N=80, test set N=20, dice score lateral ventricles 0.94±0.02, third ventricle 0.90±0.10, fourth ventricle 0.85±0.15). The codes of all models have been made available on github.com (https://github.com/jqmcginnis/ich_segmentation). Lesion masks were visually screened and 7% had to be manually corrected (with ITK-Snap). Subsequently, lesion mask volumes were computed using the affine transformations from the NIfTI headers, leveraging the Python-based Nibabel and Numpy libraries.

#### Voxel-Based Lesion-Symptom Mapping

To identify brain regions associated with central fever but not with infectious fever, we conducted a VLSM analysis^19^ with central fever as a variable of interest comparing it to cases with infectious fever. Therefore, CT scans and corresponding lesion masks of PH were warped into a 1×1×1 mm stereotaxic space using the CT-normalise function in the Statistical Parametric Mapping package (SPM, V.12).^20^ To ensure statistical power, we created a lesion frequency map, running the Python-based Matplotlib library (V.3.6.2). VLSM was performed using the NiiStat toolbox (RRID:SCR_014152, www.nitrc.org/projects/niistat), requiring MATLAB and SPM, including only voxels lesioned in at least five patients. The Liebermeister test with 8000 permutations was conducted for non-parametric mapping, applying a false discovery rate (FDR) of 5% to correct for multiple comparisons.^21^ Lesion masks and statistical maps were coregistered and warped to the MNI 152 template (Montreal Neurological Institute, voxel size of 1×1×1 mm^3^) using FSL.^22 23^ Brain regions containing significant voxels were identified using anatomical atlases. The analyses containing the assignment of voxels to atlas labels have been made available on GitHub (https://github.com/jqmcginnis/ich_central_fever). To incorporate the results of the VLSM into our multiple regression analysis, lesion masks from each patient were analysed for overlapping with significant clusters of voxels from VLSM (0: no overlap; 1: any overlap) using FSLeyes.

### Statistical analyses

Besides VLSM, statistical analyses were performed using IBM SPSS Statistics (V.29.0.1). A two-sample t-test was used for metric data, the Mann-Whitney U test for ordinal data and the χ^2^ test for binary categorical variables. Furthermore, to identify parameters that independently distinguish central from infectious fever on the day of fever onset, a binomial logistic regression analysis was conducted, including all parameters associated with central fever from univariate analyses. Multicollinearity diagnostics (tolerance value and variance inflation factor) were performed to evaluate correlations among predictor variables.

## Results

A total of 547 ICH patients were available for analysis. Fever occurred in 213 patients; 54 were diagnosed with central fever, 156 with infectious fever and three patients had causes of fever other than central or infectious (one case each of neutropenic, rheumatic and drug fever). Table 1 provides an overview of the results from univariate analyses, comparing clinical, imaging and laboratory parameters between the groups with central and infectious fever.

**Table 1:** Results of univariate analyses

	Central fever N=54	Infectious fever N=156	P value
Baseline parameters			
Age, years, mean (SD)	69.1 (±15.3)	67.4 (±14.2)	0.49
Sex, male, number (%)	33 (61)	103 (66)	0.65
Premorbid mRS, median (IQR)	1 (0–1)	1 (0–1)	0.99
Antiplatelet drugs, number (%)	13 (24)	42 (27)	0.65
Oral anticoagulants, number (%)	13 (24)	35 (22)	0.82
NIHSS, admission, median (IQR)	28 (15–36)	18 (13–28)	**0.008**
Fever characteristics			
Onset day, median (IQR)	2 (1-3)	6 (4-9)	**<0.001**
Duration of fever period, days, median (IQR)	4 (3-9)	3 (2-4)	**<0.001**
Peak temperature, °C, mean (SD)	39.3 (±0.5)	39.0 (±0.7)	**0.001**
Increase of SOFA-Score≥2, number (%)	2 (4)	51 (33)	**<0.001**
SIRS, number (%)	27 (50)	137 (88)	**<0.001**
Antibiotic treatment, number (%)	23 (43)	145 (93)	**<0.001**
Resistant to antipyretic treatment, number (%)	18 (33)	15 (10)	**<0.001**
Imaging parameters			
Deep brain ICH, number (%)	27 (50)	80 (51)	0.84
Hemisphere, left, number (%)	32 (59)	92 (59)	0.99
Overlap midbrain cluster*†(54/150), number (%)	10 (19)	1 (1)	**<0.001**
Overlap hypothalamic cluster*†(50/150), number (%)	14 (26)	6 (4)	**<0.001**
Brainstem compression, median (IQR)	1 (0–1)	0 (0–0)	**0.004**
Midline shift, mm, mean (SD)*‡,(45/120)	4.8 (±4.8)	4.1 (±3.6)	0.358
PH volume, mL, mean (SD)	54.4 (±54.4)	42.7 (±37.6)	0.15
Oedema volume, mL, mean (SD)	34.9 (±32.8)	30.7 (±26.4)	0.40
Intraventricular extension, number (%)	50 (74)	127 (81)	0.25
IVH volume, mL, median (IQR)	5.6 (0.1–27.5)	6.1 (0.5–20.8)	0.85
Lateral ventricles	3.9 (0–25.1)	12.0 (±18.1)	0.89
Third ventricle	0.0 (0–2.6)	0.1 (0–1.6)	0.92
Fourth ventricle	0.0 (0–2.8)	0.0 (0–1.6)	0.53
Laboratory parameters			
Leucocyte count, x10^3/^µl, mean (SD)			
First day of fever *(50/152)	10.0 (±3.4)	11.2 (±3.7)	**0.046**
Peak*(53/155)	13.1 (±4.2)	16.3 (±5.6)	**<0.01**
CRP, mg/dL, mean (SD)			
First day of fever*(33/86)	0.7 (±1.5)	6.4 (±6.0)	**<0.01**
Peak*(26/112)	5.3 (±5.6)	11.4 (±9.5)	**<0.01**
PCT, ng/mL, mean (SD)			
First day of fever*(41/102)	0.1 (±0.1)	1.9 (±9.3)	0.06
Peak*(41/103)	0.3 (±0.3)	4.6 (±14.1)	**0.002**
Outcome parameters			
mRS at discharge, median (IQR)	6 (4-6)	5 (4-5)	**0.006**
Mortality, number (%)	31 (57)	39 (25)	**<0.01**

* Refers to missing values, N is given in square brackets as [central fever/infectious fever].

† Midline shift was not determined in solely infratentorial ICH.

‡ Only VLSM inclusions.

CRP, C reactive protein; ICH, intracerebral haemorrhage; IVH, intraventricular haemorrhage; mRS, modified Rankin Scale; NIHSS, National Institutes of Health Stroke Scale; PCT, procalcitonin; PH, parenchymal haemorrhage; SIRS, Systemic Inflammatory Response Syndrome; SOFA, Sequential Organ Failure Assessment; VLSM, Voxel-Based Lesion-Symptom Mapping.

### Clinical and laboratory parameters

Central fever was associated with higher NIHSS scores, worse functional outcomes and higher mortality (p<0.01). Conversely, infectious fever was associated with higher CRP levels and leucocyte counts (p<0.01) on the first day of fever and at peak levels; whereas PCT was only significantly higher at peak levels.

### Imaging

Parameters from visual and quantitative image assessment revealed one major difference: the extent of brainstem compression, which was more severe in central fever compared with infectious fever. For VLSM analysis, 204 patients (54 with central, 150 with infectious fever) were included, while six patients were excluded due to unsuccessful normalisation related to the presence of only IVH without accompanying PH; figure 1 shows a lesion overlay. In VLSM, the FDR of <0.05 resulted in Z-score thresholds of <−4.5 and >3.5 and revealed 84 voxels associated with central fever (figure 2). Most of the 84 voxels were part of two larger clusters: one in the diencephalon and another in the brainstem. According to the Neudorfer *et al* high-resolution MRI atlas of the human hypothalamic region,^24^ the largest proportion of voxels (60%) was located in the left hypothalamic region, while, according to the brainstem atlas of Tang *et al*,^25^ the second cluster (18%) was located in the left midbrain. As expected, univariate analysis also showed a strong association between central fever and an overlap with either of the two clusters (table 1). Given that 9 out of 11 patients with lesions overlapping the brainstem cluster had lesions also overlapping the larger hypothalamic cluster, we only considered the latter for further analyses.

**Figure 1:**
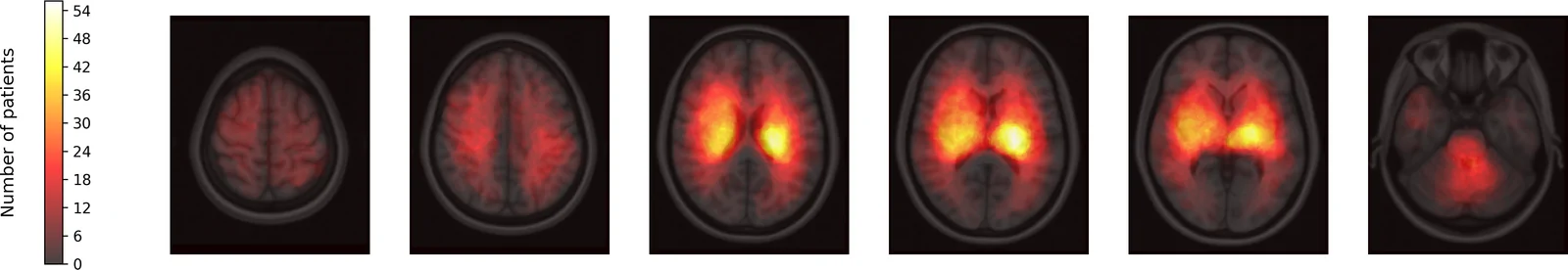
Lesion overlay of VLSM analysis in radiological convention. VLSM, Voxel-Based Lesion-Symptom Mapping.

**Figure 2:**
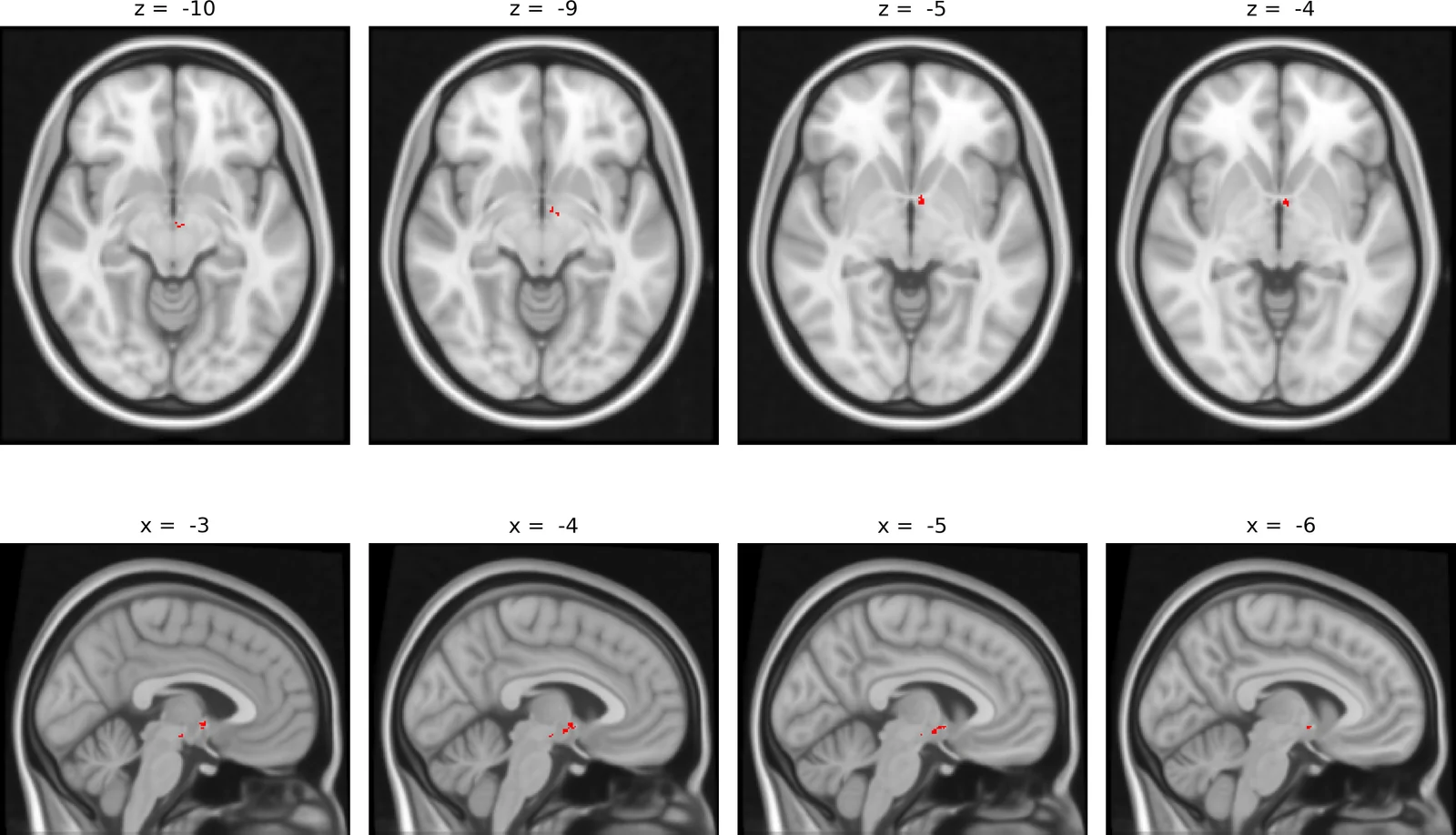
Results of VLSM in radiological convention. Voxels significantly associated with central fever are displayed in red. Given coordinates refer to MNI space. VLSM, Voxel-Based Lesion-Symptom Mapping.

### Fever characteristics

Central fever went along with higher peak temperatures, longer fever episodes and an earlier onset of fever; it was more often resistant to antipyretic treatment, whereas septic courses, according to the SIRS and SOFA criteria, were more frequent in infectious fever.

### Binomial regression analysis

Table 2 presents the results of our binomial regression model, which was based on parameters available on the day of fever onset and significantly associated with central fever in univariate analyses. To ensure statistical power, we excluded CRP levels from the first day of fever because more than 40% of the values were missing. To address a possible selection bias, we compared patients with and without available CRP; the latter were younger and more severely affected (online supplemental table 1).

**Table 2:** Results of binomial regression model

Parameter	Estimates	OR	95% CI, lower bound	95% CI, upper bound	P value
NIHSS on admission	−0.012	0.988	0.939	1.039	0.63
Overlap hypothalamic cluster	2.272	9.701	1.600	58.837	**0.01**
Day of fever onset	−1.492	0.225	0.113	0.380	**<0.001**
Leucocyte count, first day of fever	−0.256	0.774	0.656	0.913	**0.002**
Brainstem compression	−0.024	0.976	0.508	1.875	0.37

NIHSS, National Institutes of Health Stroke Scale.

With a McFadden R² of 0.57 and an area under the curve of 0.95, our model demonstrated excellent performance. Multicollinearity diagnostics indicated tolerance values between 0.73 and 0.86, and variance inflation factors between 1.17 and 1.40 for all included variables, suggesting a low degree of multicollinearity in our model. The day of fever onset emerged as the strongest independent predictor of central fever. At the same time, an overlap with the cluster in the left hypothalamic region and leucocyte counts on the first day of fever also distinguished between central and infectious fever (table 2).

### Central versus infectious fever: ORs by day of fever onset

As the day of fever onset emerged as the main differentiator between central and infectious fever, we conducted an additional analysis using the day of fever onset as an endpoint to compute ORs to illustrate probabilities of central versus infectious fever for each day. Figure 3 demonstrates that central fever is significantly more likely than infectious fever on the first 2 days of ICH, while onset of central fever after day 4 (2 of 54 cases) is rare.

**Figure 3:**
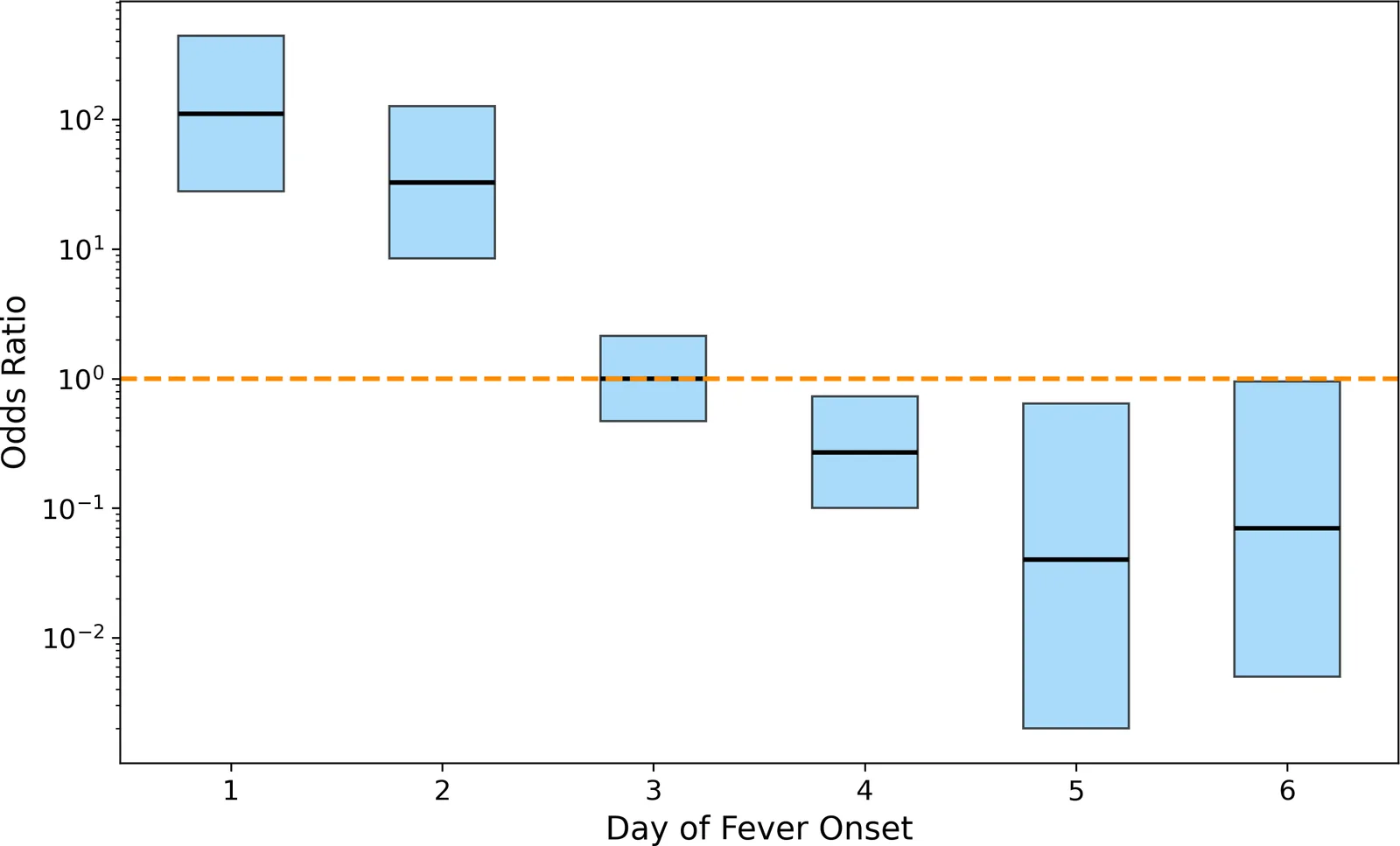
Odds ratios (ORs) comparing central and infectious fever by day of onset. Box plots show the ORs and 95% CIs comparing central to infectious fever for each day. Note the considerable changes indicated by the logarithmic scale: initially favouring central over infectious fever, which inverts abruptly on day 3.

## Discussion

We identified clinical, laboratory and imaging parameters associated with central fever that may aid in differentiating it from infectious fever in ICH. First, early onset of fever appears to be the strongest indicator of central fever. As additional factors for differentiating central from infectious fever, we found infectious parameters and lesion location. The latter may be related to the development of central fever when thermoregulation centres, especially the hypothalamic region, are affected. In these specific scenarios, there appears to be a considerable risk of overusing antibiotics, as more than 40% of patients with central fever in our cohort initially received antibiotic treatment.

The frequency of central fever in our cohort was 10%, with a ratio of central to infectious fever of 1:3, which was lower than in previous studies on ICH.^14 15^ However, we opted for more conservative criteria to ensure a higher positive predictive value: (1) we defined fever as elevated temperature on *at least 2 consecutive days*, as we believe this approach is less susceptible to false positives, particularly since central fever is known for its longer duration^6 7^ and (2) we opted for an inclusive definition of infectious fever, classifying fever as infectious in cases of doubt to minimise the risk of erroneous classification of central fever, accepting that some non-infectious conditions such as atelectasis, or thromboembolism, may have been classified as infectious fever.

The early onset of central fever emerged as the key differentiator from infectious fever. While this is the first study to demonstrate this finding in a cohort solely of ICH patients, the early onset of central fever is well known in other disorders, such as subarachnoid haemorrhage.^6 7^ We hypothesise that when an ICH capable of causing central fever occurs, the latency between the stroke and central fever onset is relatively short—typically within, or soon after, the period of maximum haematoma expansion, usually within 24 hours.^26–28^ In contrast, infectious fever reflects a complication of ICH, most likely as a symptom of pneumonia or urinary tract infections, usually developing over days with a start later than day 3 (figure 3).

The value of infectious parameters in differentiating central fever from infectious fever is controversial in the literature.^6 15 29 30^ Additionally, to the best of our knowledge, there are only sparse data on infectious parameters from the first day of fever reported in previous studies,^6 14 15^ well in line with the fact that also in our cohort CRP and PCT values from the first day of fever were incomplete. Although we found significant differences in peak leucocyte count, CRP and PCT levels between central and infectious fever groups and identified lower leucocyte counts on the day of fever onset as a potential indicator of central fever, CIs still largely overlapped, hampering reliable conclusions solely from these parameters in the individual patient.

Applying VLSM analyses, we identified voxels primarily in the left hypothalamic region associated with central fever. Involvement of this region emerged as an independent predictor of central fever in our logistic regression analysis. This area is linked to thermoregulation and has previously been connected to central fever.^31^ Although functional lateralisation of the hypothalamic region has been observed in animal models, we are not aware of evidence for lateralisation of thermoregulation in humans that would explain our lateralised finding.^32–34^ Moreover, since there was a slight predominance of left hemispheric ICH in patients with fever in our cohort, the statistical power for the right hemispheric hypothalamic region may have been lower (figure 1). Therefore, we cannot definitively conclude on the role of the right hypothalamic region in central fever.

We acknowledge the limitations of our study. In addition to the retrospective and single-centre study design, our data, especially CRP and PCT levels on the first day of fever, were partially incomplete. Moreover, we identified a potential selection bias related to CRP, which is routinely used for evaluating infection diagnoses in our stroke unit, whereas our ICU typically relies on other infectious parameters such as leucocyte counts, PCT and interleukin-6. Consequently, patients with missing CRP values represent a cohort with more severe strokes and higher mortality (online supplemental table 1). Although our sample size was relatively large, our findings need replication in an independent cohort. Future analyses may define cut-off values useful at the individual patient level. Additionally, although infectious biomarkers were not a primary criterion in the retrospective evaluation of infectious diagnosis,^16^ we acknowledge that they may have influenced initial clinical decisions. Moreover, VLSM was based on CT scans with limited diagnostic accuracy in regions containing the majority of significant voxels, that is, brainstem and hypothalamus.^35 36^ Additionally, the significant deformity of brains in some patients may have compromised spatial normalisation.^37^

Additionally, due to our definition of fever and inclusion criteria, some cases of infectious and central fever—particularly those with a shorter duration of fever episodes or rapid fatal courses within 48 hours—may have been missed. Finally, although we chose conservative criteria for central fever, some patients may have been misclassified, given the lack of comprehensive and largely accepted diagnostic criteria.

In summary, we have identified the day of onset and involvement of the left hypothalamic region as the strongest indicators of central fever in patients with ICH. Our findings may contribute to developing evidence-based treatment decisions for patients with fever in ICH and potentially optimise antibiotic stewardship.

## Supplementary material

10.1136/svn-2025-004441 online supplemental file 1

## Data Availability

Data are available upon reasonable request.
