# Supplementary material for: Differentiating central fever from infectious fever in intracerebral haemorrhage
Source: Stroke Vasc Neurol. 2025 Aug 27;11(5):e004441. doi: 10.1136/svn-2025-004441 (PMC13728880; doi:10.1136/svn-2025-004441)
Supplement: Supplementary file 1 [file svn-11-5-s001.pdf]

Supplementary Figure 1: Dropout Flowchart

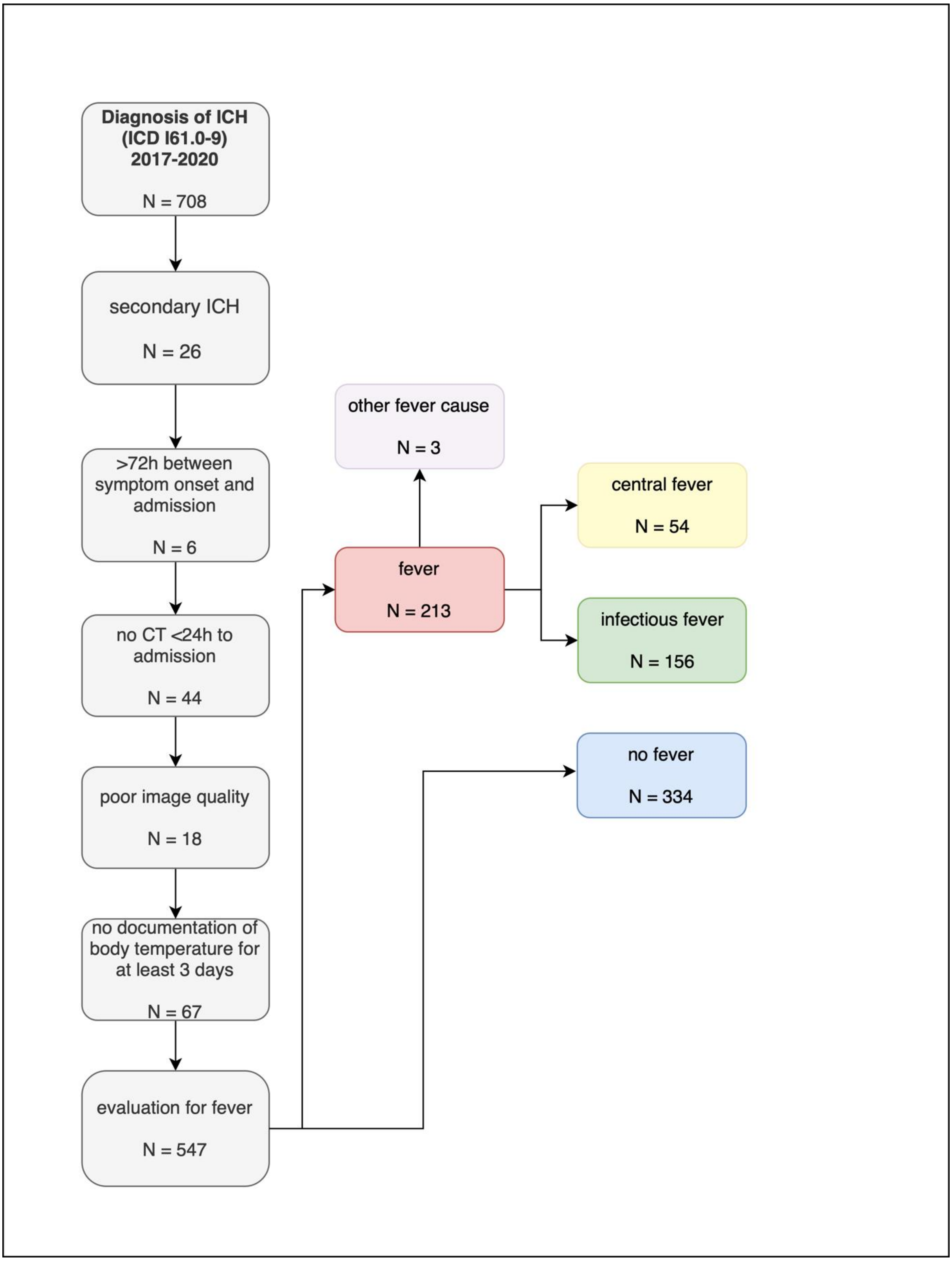

ICH = intracerebral hemorrhage, CT = computed tomography, secondary ICH refers to ICH caused by vascular malformations, neoplasms, or hemorrhagic transformation of ischemic stroke

Supplementary Table 1: Baseline data of patients with and without CRP on the first day of fever.

	**CRP available N = 117**	**CRP not available N = 89**	**P value**
**Baseline parameters**			
Age, y, mean (SD)	70.8 (±13.3)	64.2 (±15.4)	**<0.01**
Sex, male, No. (%)	75 (64)	42 (47)	0.50
Premorbid mRS, median (IQR)	1 (0-1) <	1 (0-1)	**0.04**
Antiplatelet drugs, No. (%)	38 (32)	18 (20)	**0.03**
Oral anticoagulants, No. (%)	29 (25)	19 (21)	0.54
NIHSS, admission, median (IQR)	17 (10-28)	26 (18-33)	**<0.01**
**Fever Type**			
Central (vs. infectious)	32 (1-3)	22 (4-9)	0.67
**Imaging parameters**			
Deep brain ICH, No. (%)	74 (63)	46 (52)	0.08
Hemisphere, left, No. (%)	57 (49)	47 (53)	0.51
PH volume, mL, mean (SD)	43.2 (±43.1)	48.8 (±42.7)	0.82
Edema volume, mL, mean (SD)	32.3 (±28.4)	31.3 (±26.7)	0.40
Presence of IVH, No. (%)	86 (74)	78 (88)	**0.01**
IVH volume, mL, median (IQR)	15.5 (26.3)	16.8 (22.1)	0.65
**Outcome parameters**			
Mortality, No. (%)	32 (27)	37 (42)	**0.03**

Abbreviations: SD = standard deviation, IQR = interquartile range, NIHSS = National Institutes of Health Stroke Scale, mRS = modified Rankin Scale, PH = parenchymal hemorrhage, IVH = intraventricular hemorrhage, CRP = c-reactive protein.
